# Interdependency of selected metabolic variables in an animal model of metabolic syndrome

**DOI:** 10.3892/etm.2014.1886

**Published:** 2014-08-06

**Authors:** ZOHEIR MELLOUK, ABDULLAH SENER, DALILA AIT YAHIA, WILLY J. MALAISSE

**Affiliations:** 1 Department of Biology, Es-Sénia University, Oran, Algeria; 2 Laboratory of Experimental Hormonology, Université Libre de Bruxelles, Brussels, Belgium

**Keywords:** metabolic syndrome, fructose-fed rats, long-chain polyunsaturated ω3 and ω6 fatty acids

## Abstract

In the present study, the correlation between the percentage of glycated hemoglobin, taken as representative of changes in glucose homeostasis, and selected variables was investigated. Rats were treated for 8 weeks with diets containing 64% starch and 5% sunflower oil or containing 64% D-fructose mixed with: 5% sunflower oil; 3.4% sunflower oil and 1.6% salmon oil; or 3.4% sunflower oil and 1.6% safflower oil. Positive correlations were found between glycated hemoglobin and plasma albumin, urea, creatinine, phospholipids, triglycerides and total cholesterol, liver cholesterol, triglyceride and phospholipid content, and the plasma, liver, heart, kidney, soleus muscle and visceral adipose tissue content of thiobarbituric acid reactive substances, carbonyl derivatives and hydroperoxides. Inversely, negative correlations were observed between glycated hemoglobin and plasma calcium, iron and HDL-cholesterol concentrations, liver, heart, kidney, soleus muscle and visceral adipose tissue superoxide dismutase and catalase activity; as well as plasma, liver, heart, kidney, soleus muscle and visceral adipose tissue nitric oxide content. Only the liver glucokinase activity and liver, heart, kidney, soleus muscle and visceral adipose tissue glutathione reductase activity failed to display a significant correlation with glycated hemoglobin. These findings confirm the hypothesis that there is a close association between glucose homeostasis and other variables when considering the effects of long-chain polyunsaturated ω3 and ω6 fatty acids in rats with fructose-induced metabolic syndrome.

## Introduction

As previously reviewed (1), the induction of metabolic syndrome in rats by a fructose-rich diet has been, over recent years, the subject of several extensive investigations (2–10). The present study is founded on an extensive investigation of the post-mortem findings collected from rats exposed for 8 weeks to diets containing 64% starch and 5% sunflower oil (Ssun rats) or containing 64% D-fructose mixed with 5% sunflower oil (Fsun rats), 3.4% sunflower oil and 1.6% salmon oil (Fsal rats) or 3.4% sunflower oil and 1.6% safflower oil (Fsaf rats) (8–10). The major aim of the present study was to explore the possible correlation, at the individual level, between the percentage of glycated hemoglobin, considered as indicative of glucose homeostasis, and 55 other variables measured in plasma, liver, heart, kidney, soleus muscle or visceral adipose tissue. For each of these variables, individual data were collected from 20–23 animals.

## Materials and methods

### Biological variable analysis

The methods used to measure glycated hemoglobin (5), plasma albumin, urea, creatinine, calcium, iron, phospholipid, triglyceride and HDL-cholesterol (8), hepatic glucokinase activity and liver cholesterol, triglyceride and phospholipid content (8), systolic arterial blood pressure (9), plasma leptin concentration (9), kidney proliferating cell nuclear antigen index (9), liver, heart, kidney, soleus muscle or visceral adipose tissue glutathione reductase, superoxide dismutase and catalase activity (9), plasma, liver heart, kidney, soleus muscle or visceral adipose tissue thiobarbituric acid reactive substances, carbonyl derivatives, hydroperoxides and nitric acid content (10) were previously described in the references cited. This study was approved by the ethics committee of the Université Libre de Bruxelles.

### Correlation analysis

In the correlation data, the xy values refer to the product of the differences, at the individual level and for the two variables under consideration, between the recorded individual measurement and the corresponding mean value. The correlation analysis was performed by W.J. Malaisse, one of the authors of the present article, without the use of any software.

## Results

### Correlation between glycated hemoglobin and selected plasma variables

The percentage of glycated hemoglobin was considered to be indicative of glucose homeostasis. Table I presents the results of a correlation analysis between glycated hemoglobin and nine plasma variables. Significant positive correlations were found between glycated hemoglobin and plasma albumin, urea, creatinine, phospholipids, triglycerides and total cholesterol concentrations, whilst negative correlations were found between glycated hemoglobin and plasma calcium, iron and HDL-cholesterol concentrations. In several cases with positive correlations, no negative xy product (two cases), or only two negative xy products (two cases) were recorded among the 23 xy products.

### Correlation between glycated hemoglobin and selected liver variables

The results from the correlation analysis between glycated hemoglobin percentage and selected liver variables are presented in Table II. No significant correlation was observed between the percentage of glycated hemoglobin and hepatic glucokinase activity. However, a significant positive correlation was observed between glycated hemoglobin and liver cholesterol, triglyceride and phospholipid content, with either no negative xy product (two cases) or only one negative xy product (one case).

### Correlation between glycated hemoglobin and blood pressure, leptin and kidney proliferating cell nuclear antigen index

A significant positive correlation (r=+0.9127; n=23; P<0.001) was observed between the percentage of glycated hemoglobin and the incremental area under the curve associated with changes in systolic arterial blood pressure as a function of time over a period of 56 days. Similarly, a highly significant positive correlation was found between glycated hemoglobin and plasma leptin concentration (r=+0.9890; n=23; P<0.001), without a negative xy product. The correlation coefficient between glycated hemoglobin and the kidney proliferating cell nuclear antigen index was +0.5589 (n=23), yielding P<0.008.

### Correlation between glycated hemoglobin and selected enzymatic activities

As shown in Table III, no significant correlation was identified between glycated hemoglobin percentage and the activity of glutathione reductase in the liver, heart, kidney, soleus muscle or visceral adipose tissue. With the exception of the adipose tissue, there was a trend towards a negative correlation. However, even when the enzymatic measurements obtained from the liver, heart and kidney were expressed relative to the mean corresponding value recorded in Ssun rats, the correlation coefficient between the normalized values and those concerning glycated hemoglobin were not statistically significant (r=−0.2148; n=69; P<0.08).

By contrast, highly significant negative correlations (P<0.001) were identified in all five organs between the percentage of glycated hemoglobin and the activity of superoxide dismutase (Table III). Significant negative correlations (P<0.05 or less) were also observed in these five organs between the individual values for glycated hemoglobin percentage and catalase activity (Table III).

### Correlation between glycated hemoglobin and selected metabolites

Positive correlations were observed between the content of thiobarbituric acid reactive substances (TBARS), carbonyl derivatives and hydroperoxides in the plasma, liver, heart, kidney, soleus muscle and visceral adipose tissue, on one hand, and glycated hemoglobin, on the other, such positive correlations only failing to achieve statistical significance in two out of eighteen comparisons (Table IV). Likewise, with the exception of the soleus muscle, highly significant negative correlations (P<0.001) were observed between glycated hemoglobin percentage and the nitric oxide (NO) content of plasma, liver, heart, kidney or visceral adipose tissue (Table IV).

## Discussion

Only two variables failed to display a significant correlation with the percentage of glycated hemoglobin. One of these was liver glucokinase activity. The only difference between the four groups of rats with regard to liver glucokinase activity was that higher activity was observed in the Fsal rats compared with the Ssun, Fsun or Fsaf rats (8). Therefore, it is hypothesized that this difference may be attributed to the greater supply of long-chain ω3 fatty acids in the diet of the Fsal rats compared with that in the diets of the other three groups of rats (8). In addition, no significant correlations were observed between glycated hemoglobin percentage and the enzyme activity of glutathione reductase in the liver, heart, kidney, soleus muscle and visceral adipose tissue. In these organs, the trend was towards a negative correlation.

A positive correlation with glycated hemoglobin percentage was observed for the plasma albumin concentration. Since an opposite situation could speculatively be expected from either liver dysfunction or renal albuminuria in the rats with severe metabolic syndrome, this positive correlation may reflect a modest degree of hemoconcentration in these rats. The positive correlations observed for glycated hemoglobin percentage with plasma urea and creatinine may reflect incipient nephropathy (8).

In terms of the cause-and-effect relationship, the negative correlations observed for glycated hemoglobin percentage with plasma calcium or iron concentrations remain unclear. They may, as discussed previously (8), be attributed to impaired gastrointestinal absorption of these metals.

The correlation between the percentage of glycated hemoglobin and the plasma concentration or liver content of cholesterol, triglycerides and phospholipids invariably yielded significant positive correlations, whereas a negative correlation was observed in the case of plasma HDL-cholesterol. These results demonstrate the tight correlation between perturbations of glucose homeostasis and the plasma concentrations or liver content of distinct types of lipids.

Furthermore, although glutathione reductase activity had no correlation with glycated hemoglobin concentration, the activity of superoxide dismutase or catalase in liver, heart, kidney, soleus muscle and visceral adipose tissue, as well as the plasma concentration or tissue content of TBARS, carbonyl derivatives and hydroperoxides were all correlated with the measurements of glycated hemoglobin, with only one clear exception (P>0.1) out of 28 comparisons. The negative correlations found for superoxide dismutase and catalase coincided with positive correlations in the case of variables indicative of oxidative stress, including the accumulation of TBARS, carbonyl derivatives and hydroperoxides.

Furthermore, the NO content of plasma, liver, heart, kidney or adipose tissue, but not that of soleus muscle, was negatively correlated with the glycated hemoglobin percentage.

In conclusion, without prejudging the cause-and-effect link between distinct variables, the present study documents the tight coordination in the metabolic, enzymatic and functional response to changes in the dietary supply of long-chain polyunsaturated ω3 and ω6 fatty acids to rats displaying a fructose-induced metabolic syndrome. All the correlations between the 55 variables under consideration were calculated by reference to the measurements of glycated hemoglobin. Hence, the latter measurements appear as most appropriate to judge the severity of biochemical and biophysical alterations in this animal model of the metabolic syndrome.

## Figures and Tables

**Table I:** Correlation between glycated hemoglobin percentage and selected plasma variables.

Variable	Correlation
Albumin	+0.9111 (P<0.001)
Urea	+0.7378 (P<0.001)
Creatinine	+0.8360 (P<0.001)
Calcium	−0.4709 (P<0.03)
Iron	−0.5008 (P<0.02)
Phospholipids	+0.8680 (P<0.001)
Triglycerides	+0.9125 (P<0.001)
Total cholesterol	+0.7837 (P<0.001)
HDL-cholesterol	−0.5431 (P<0.009)

HDL, high density lipoprotein.

**Table II:** Correlation between glycated hemoglobin percentage and selected liver variables.

Variable	Correlation
Glucokinase activity	−0.0781 (P>0.1)^a^
Cholesterol content	+0.9310 (P<0.001)
Triglyceride content	+0.9962 (P<0.001)
Phospholipid content	+0.9818 (P<0.001)

a n=22; n=23 in all other cases.

**Table III:** Correlation between glycated hemoglobin percentage and selected enzymatic activities.

Organ	Glutathione reductase	Superoxide dismutase	Catalase
Liver	−0.2094 (P>0.1)	−0.8067 (P<0.001)	−0.4869 (P<0.03)
Heart	−0.1799 (P>0.1)	−0.9169 (P<0.001)	−0.4809 (P<0.03)
Kidney	−0.3768 (P<0.08)	−0.7388 (P<0.001)	−0.4282 (P<0.05)
Soleus muscle	−0.1428 (P>0.1)^a^	−0.9115 (P<0.001)	−0.4546 (P<0.04)
Adipose tissue	+0.2449 (P>0.1)	−0.9405 (P<0.001)	−0.5616 (P<0.008)

a n=20; n=23 in all other cases.

**Table IV:** Correlation between glycated hemoglobin percentage and selected metabolites.

Organ	TBARS	Carbonyls	Hydroperoxides	NO
Plasma	+0.9451 (P<0.001)	+0.6655 (P<0.001)	+0.8774 (P<0.001)	−0.9047 (P<0.001)
Liver	+0.7318 (P<0.001)	+0.7765 (P<0.001)	+0.6555 (P<0.001)	−0.6648 (P<0.001)
Heart	+0.6550 (P<0.001)	+0.6259 (P<0.003)	+0.7042 (P<0.001)^a^	−0.7585 (P<0.001)
Kidney	+0.7136 (P<0.001)	+0.3828 (P<0.08)	+0.4899 (P<0.03)	−0.8589 (P<0.001)
Soleus muscle	+0.7619 (P<0.001)	+0.5864 (P<0.006)	+0.8287 (P<0.001)	+0.4761 (P<0.03)
Adipose tissue	+0.8343 (P<0.001)	+0.4820 (P<0.03)	+0.3220 (P>0.1)	−0.8369 (P<0.001)

a n=21; n=23 in all other cases.

TBARS, thiobarbituric acid reactive substances; NO, nitric oxide.
